# Anatomical study between the correlation of the arcuate eminence and the superior semicircular canal

**DOI:** 10.1007/s00276-021-02834-2

**Published:** 2021-09-20

**Authors:** A. García-Barrios, A. I. Cisneros, J. Obon, R. Crovetto, J. Benito, J. Whyte

**Affiliations:** 1 Department of Human Anatomy and Histology, School of Medicine, University of Zaragoza, C/ Domingo Miral, s/n, 50009 Zaragoza, Spain; 2 Medical and Genetic Research Group (GIIS099) IIS Aragón, Zaragoza, Spain; 3 Bilbao Clinic, Bilbao, Spain

**Keywords:** Arcuate eminence, Superior semicircular canal, Computed tomography

## Abstract

**Objective:**

To study the anatomical correlation between the arcuate eminence and the superior semicircular canal.

**Material and methods:**

A study of the height of the arcuate eminence was carried out in 295 temporal bones. In addition, 30 temporals with different heights of the arcuate eminence (10 flat, 10 prominent and 10 very prominent) were randomly selected and radiological tests were performed by computed tomography (Pöschl projection) and subsequent dissection by milling until the apex of the superior semicircular canal was found, establishing, with both methods, the anatomical relationship with the arcuate eminence.

**Results:**

The arcuate eminence was classified as: smooth, when there was no relief (1.7%); flat, measured less than 1 mm (20.3%), prominent, measured between 1 and 2 mm, in (62%), and very prominent, measured above 2 mm (12.6%). The tomographic study (CT) and its subsequent dissection by bone milling showed a direct relationship between the arcuate eminence and the semicircular canal only when it was flat, while the rest of the types corresponded to the presence of pneumatized peri-labyrinthine cells and/or cancellous bone without a direct anatomical relationship with the apex of the superior semicircular canal.

**Conclusion:**

The correlation between the arcuate eminence and the superior semicircular canal is direct only when it is flat (1 mm), being related to peri-labyrinthine cells and/or cancellous bone when the arcuate eminence is prominent or very prominent.

## Introduction

In the more classical treatises on human anatomy, the arcuate eminence is defined as the elevation produced by the prominence of the superior semi-circular canal on the anterior surface of the temporal bone, at the junction of the posterior third with the anterior two thirds, whereby it is related to the middle cranial fossa, and separated from it by the meninges [1, 2]. It is used in surgery of the middle cranial fossa as a reference point to locate the internal acoustic canal within the temporal bone [3–5]. However, there are studies in which this eminence does not coincide exactly with the superior osseous semi-circular canal, but adapts to the occipitotemporal sulcus on the inferior surface of the temporal lobe of the cerebral hemisphere [6, 7]. There are others in which the arcuate eminence is caused by pneumatization processes of the epitympanic region [8, 9].

The aim is to study the anatomical morphology of the arcuate eminence and its relationship with the superior semicircular canal by radiological study and subsequent anatomical dissection, after milling, and to assess the correlation between the two structures.

## Materials and methods

The morphological study of the arcuate eminence was carried out on 295 pieces from the ossuary (75 skulls from which the calotte had been previously cut, and 145 isolated temporal bones) belonging to the Department of Human Anatomy and Histology in the School of Medicine (University of Zaragoza).

The arcuate eminences were classified in four groups: smooth (without relief), flat (less than 1 mm), prominent (1–2 mm) and very prominent (greater than 2 mm), according to a measurement made by means of a digital caliper taking as reference the lowest and highest points of the Arcuate Eminence.

In addition, CT radiological tests were randomly performed in 30 of the specimens reviewed: 10 with flat eminence, 10 with prominent eminence and 10 with very prominent eminence. The radiological study was not considered in the smooth eminences because they did not show any relief. Subsequently, the arcuate eminence was reamed in its most prominent area, extending as far as necessary to locate the superior semicircular canal, establishing the anatomical relationship between both structures.

The radiological studies was performed by means of multi-splice helical computed tomography equipment (Philips Brilliance 6).

For image acquisition and formatting, the radiological protocols were follows:

2 × 0.6 mm collimation, 0.65 mm splice thickness, 0.32 mm splice increase, 0.75 s rotation time, 0.38 piCTh, 120CV, 300 mAs, 1024 × 1024 matrix, 180 mm field of vision, 0.5 mm reconstruction thickness and 0.5 mm reconstruction increase.

In each temporal bone, both coronal and Pöschl plane reconstructions were carried out. The “raw data” have been reconstructed using a bone algorithm. The relationship between the apex of the superior semicircular canal and the most prominent region of the arcuate eminentia was established in successive radiological sections of each temporal studied.

## Results

### (1) Ossuary material

**Fig. 1:**
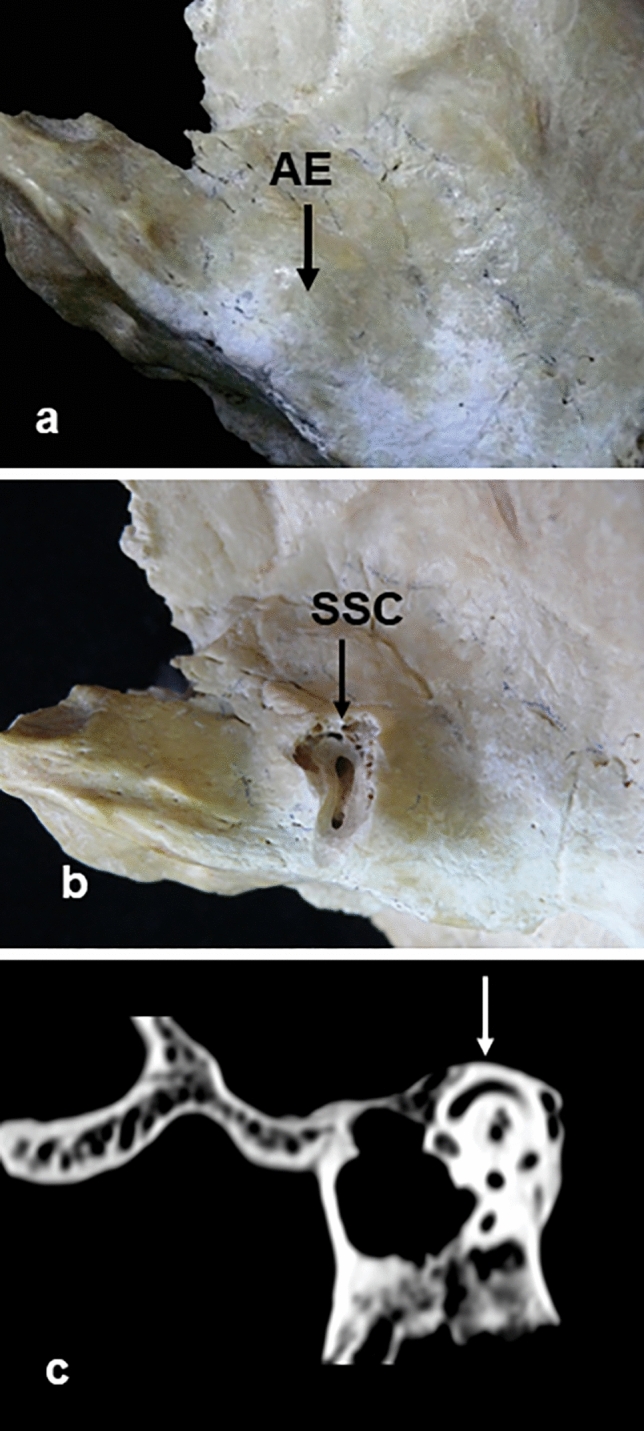
Note in **a** the presence of arcuate eminence flat type, in **b** how the milling reveals its correspondence with the superior semicircular canal, and in **c** tomographic image of such evidence. *AE* arcuate eminence, *SSC* superior semi-circular canal. Arrow: arcuate eminence relief

**Fig. 2:**
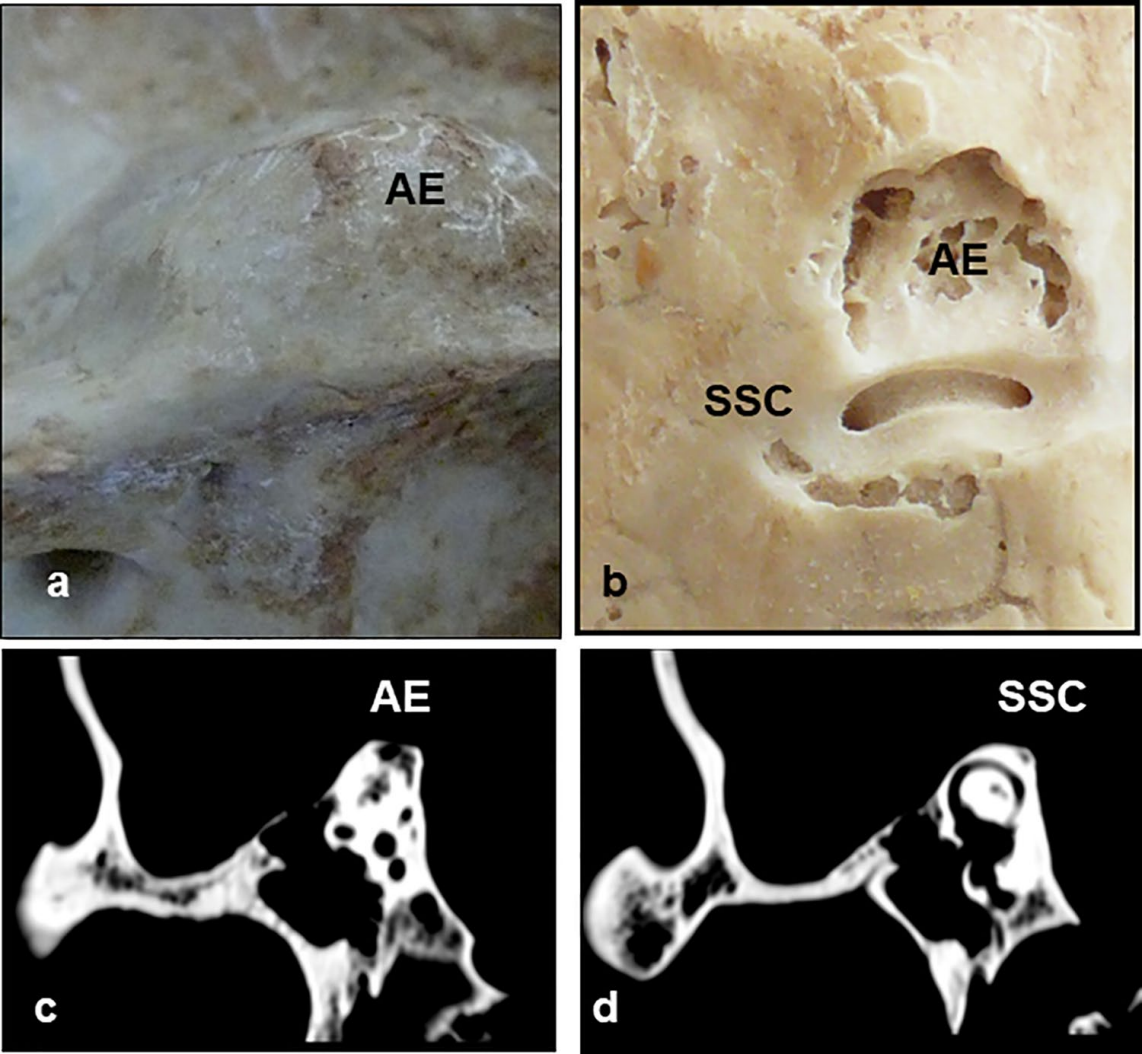
See in **a** the presence of arcuate eminence prominent type, in **b**, how the milling of the AE reveals supra-labyrinthine cells and the superior semicircular canal (SSC), remaining anterior and medial to it. Computed Tomography shows how the arcuate eminence corresponds to supra-labyrinthine cells **c** and in **d** the superior semi-circular canal. *AE* arcuate eminence, *SSC* superior semi-circular canal

**Fig. 3:**
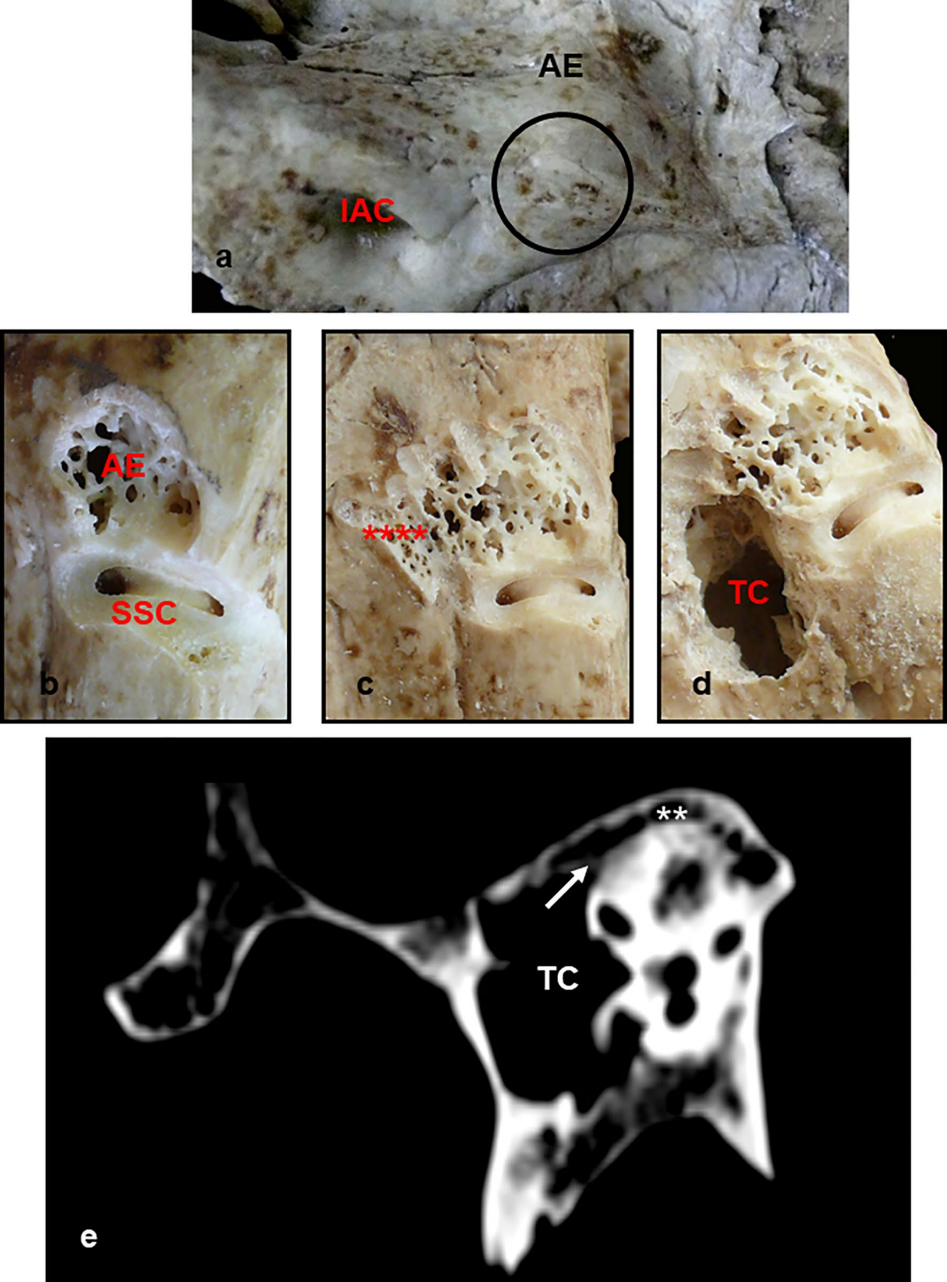
Note in **a** the presence of arcuate eminence very prominent and in **b**, how the milling corresponds to supra-labyrinthine cells, and the superior semicircular canal lies antero-medially to this. In **c**, **d** y **e**, as these cells originate from the attic of the tympanic cavity. *AE* arcuate eminence, *IAC* internal auditory canal, *SSC* superior semi-circular canal, **** Tegmen tympani, *TC* tympanic cavity; Arrow: prolongation of cells from the attic; ** pneumatic cells

In the macroscopic study of the osseous pieces, the arcuate eminence was classified into four types: smooth, when no relief was visible, in five of the pieces (1.7%); flat, measuring less than 1 mm, in 60 of the pieces (20.3%) (Fig. 1a); prominent, measuring between 1 and 2 mm, in 183 pieces (62%) (Fig. 2a); and very prominent, measuring more than 2 mm, in 37 pieces (12.6%) (Fig. 3a).

On the other hand, 10 of the pieces (3.4%) were discarded from the study due to dehiscence of the semi-circular canal (2/295), or breakage of the petrous bone (8/295).

### (2) Tomographic study

In the tomographic studies carried out, it was observed that in the pieces where the arcuate eminence was flat (less than 1 mm), the different reconstruction cuts in the canal plane showed that the arcuate eminence corresponded to the relief left by the superior semi-circular canal on the antero-superior surface of the osseous crest (Fig. 1c).

However, when the Arcuate Eminence was prominent, between 1-2 mm (Fig. 2c, d) or very prominent, above 2 mm (Fig. 3e), no concordance was observed between it and the superior semicircular canal, the arcuate eminence corresponding to supra-labyrinthine cells and/or non-compacted bone, and the superior semi-circular canal becoming visible in later sections. The cells were present in 74.6%, the rest was cancellous bone.

3) Dissection of the arcuate eminence.

After anatomical dissection by milling of the selected pieces, it was observed that in the 10 pieces with flat arcuate eminence, there was a direct correspondence with the superior semi-circular canal (Fig. 1b).

In the other 20 pieces, 10 with prominent arcuate eminence, and 10 with very prominent arcuate eminence, a direct relationship with peri-labyrinthine cells was seen. This was much more obvious in the pieces with very prominent arcuate eminence. In these 20 bones, more extensive milling was performed, showing that, in all of them, the location of the superior semi-circular canal was anterior and medial to the arcuate eminence (Figs. 2b, 3b–d).

## Discussion

Several hypotheses have been put forward in terms of the origin of the arcuate eminence. One of them suggests that it originates from the protrusion of the superior semi-circular canal [8], while others do not find a direct coincidence between the two structures, suggesting that the arcuate eminence may be due to pneumatization processes, or to the grooves that the temporal lobe of the brain leaves on the crest [9, 10]. In our anatomical study, with radiological tests and subsequent dissection, we found that, in the prominent or very prominent Arcuate Eminence (1–2 mm and > 2 mm, respectively), corresponding to 74.6% of the eminences studied, the relief was due to pneumatization of this region, and in the flat ones (< 1 mm), it was due to protrusion of the semi-circular canal. These results are in agreement with those of Djallian [8], who stated that, in 79.6% of the arcuate eminences studied, the highest point corresponded to pneumatic cells, located above the semi-circular canal, as well as with the data described by Shoman [10], who observed pneumatization of the arcuate eminence area in 73% of the cases.

Based on the results obtained, we believe that the arcuate eminence corresponds, in some cases, to the prominence of the superior semi-circular canal, and in others, to the presence of pneumatic cells in the superior region of the petrous portion.

When there is full coincidence between both structures, in the ontogenetic development of this region [11], it is observed that the surface or outer covering of the canal has clearly developed, adopting its characteristic shape, its convexity being orientated towards the middle cranial fossa from 8-weeks’ development. Therefore, the highest point and this protrusion form the arcuate relief. In the late stages of development and early postnatal years, the bone covering the superior semi-circular canal is the only relief visible on the petrous apophysis towards the middle cranial fossa, a fact that could explain this concordance.

The pneumatization of the eminence also has an ontogenetic explanation, since Fraile et al. [12] demonstrated that the origin of the tegmental extension of the tegmen tympani and the superior semi-circular canal is the same, the otic capsule. Moreover, they have the same type of ossification and share the same type of structure. Therefore, it is logical to believe that when peri-labyrinthine pneumatization begins, if it appears, originating either from the mastoid cells or from the tympanic box itself, in the first year of postnatal life, due to the replacement of cancellous bone with pneumatized cells, these begin to extend and progress above the labyrinth towards the outer surface of the petrous apophysis, marking the relief of the arcuate eminence. The latter will be more evident the greater the extension and quantity of cells present.

On the other hand, the progressive growth of the temporal bone causes the canal to become deeper and deeper, and below the eminence, being displaced in an antero-medial direction with respect to it.

This study has allowed us to make an original and objective classification of the morphology of the arcuate eminence according to its elevation, into smooth (no noticeable relief), flat (< 1 mm), prominent (between 1 and 2 mm) or very prominent (> 2 mm), not previously described in the literature consulted. Other classifications have been described [13, 14] in which the arcuate eminence is described as linear, flat, globular, generally balloon-like, and quadrangular, guided solely by its appearance, not having any well-defined objective criteria to designate each of the forms. This can generate controversy between subjects when classifying the same arcuate eminence.

In our study, the arcuate eminence has been related to the superior semi-circular canal in 20.3% of cases, and always when the arcuate eminence is classified as flat (less than 1 mm), while when the arcuate eminence is classified as prominent (74.6%), these relationships are established with labyrinthine cells or cancellous bone, and therefore we have found no correlation with the canal. These data are in agreement with previous studies carried out by Tsunoda [6, 7], who observed an exact coincidence of these two structures in 20% of the cases studied, without analyzing the pneumatization of the area [1, 2, 6, 7]. On the other hand, Faure [15] initially observed that the arcuate eminence and the superior semi-circular canal coincided in 37% of cases, but of these, the eminence was pneumatized in 14% of cases, and in 23% it was not. These results are similar to those obtained in our study, where the correlation is complete in cases of flat arcuate eminence without the presence of pneumatization. We thus believe that in 14% of Faure's cases, the semi-circular canal would be pneumatized, characterized by multiple supra-labyrinthine cells in the roof of the canal. Other computed tomography studies by Bulsara [16], Seo [17], Kartush [18] and Santos [19] conclude that the arcuate eminence is not a constant and reliable reference point for identifying the exact position of the superior semi-circular canal, as there is not always a direct correlation between the two.

In contrast, we do not agree with the work of Singh [14], who observed a correlation of 93%, both concordant and ambiguous, between the semi-circular canal and the arcuate eminence, as it is based on angulation (< 10° and between 10 and 45°, respectively), and these data can lead to errors when spatially relating both structures, if the objective morphological criteria, such as those we propose, are not taken into account.

In the cases where we found no concordance, we observed an almost identical medial and anterior deviation of the superior semi-circular canal with respect to the arcuate eminence, as also observed by Kartush, Seo and Nourbakhsh. [17, 18, 20].

## Conclusion

The arcuate eminence shows a direct relationship with the superior semi-circular canal when it is flat, whereas, when its morphology is prominent there is no such correlation, and the eminence is due to the presence of peri-labyrinthine cells and/or cancellous bone, with no direct anatomical relationship with the apex of the superior semi-circular canal.
